# Safety and Efficacy of Coils in Conjunction With the Pipeline Flex Embolization Device for the Treatment of Cerebral Aneurysms

**DOI:** 10.3389/fneur.2021.651465

**Published:** 2021-10-25

**Authors:** Qianqian Zhang, Qiuji Shao, Kaitao Chang, Hongyun Zhang, Yingkun He, Hugo Andrade-Barazarte, Zhiyuan Sheng, Xiao Mo, Ajmal Zemmar, Li Li, Tianxiao Li

**Affiliations:** ^1^Department of Interventional Neuroradiology, Henan Provincial People's Hospital, Zhengzhou University People's Hospital, Henan University People's Hospital, Zhengzhou, China; ^2^Henan International Joint Laboratory of Cerebrovascular Disease, Henan Engineering Research Center of Cerebrovascular Intervention, Henan Provincial People's Hospital, Zhengzhou, China; ^3^Department of Neurosurgery, Henan Provincial People's Hospital, Henan University People's Hospital, Henan University School of Medicine, Zhengzhou University People's Hospital, Zhengzhou, China; ^4^Department of Neurosurgery, Zhengzhou University People's Hospital, Henan Provincial People's Hospital, Zhengzhou, China; ^5^Beijing Key Laboratory of Fundamental Research on Biomechanics in Clinical Application, School of Biomedical Engineering, Capital Medical University, Beijing, China

**Keywords:** cerebral aneurysms, flow diverter (FD), coil, occlusion, safety and efficacy

## Abstract

**Background:** Flow diverters (FD) have shown promising results in the treatment of intracranial aneurysms (IAs). However, there is still controversy whether pipeline flex embolization device (PED flex)-assisted coils can facilitate the curing of aneurysms. Our aim was to assess the safety and effectiveness of PED flex adjunctive with coils (PED flex + coil) in the treatment of IAs.

**Method:** Patients who underwent PED flex treatment in combination with coiling between January 2018 and June 2020 were included in this study. The clinical and radiographic characteristics before and after treatments were retrospectively evaluated. The study cohort comprised of 125 patients with 140 IAs, which was subdivided into two subgroups: one group included patients treated only through PED alone, and the other group included patients treated through PED flex adjunctive with coil. Patient baseline characteristics, aneurysm characteristics, treatment-related factors, and outcomes were analyzed to determine the effectiveness of both techniques.

**Results:** Aneurysms in the PED flex + coil group were larger (10.0 ± 5.8 mm, *P* < 0.001) and wider (7.2 ± 4.6 mm, *P* = 0.002) compared with those in the PED flex group. There was no statistical difference in the perioperative complication rate between the two groups. The overall complete occlusion rate was 75.7% at 6.2 months, with 71.7% at 6.2 ± 1.7 months in the PED flex group and 85.4% at 6.2 ± 1.8 months in the PED flex + coil group, respectively. A higher percentage of satisfactory angiography results was found in the PED flex + coil group during follow-up (92.7 vs. 78.8%, *P* = 0.047).

**Conclusion:** PED flex placement with adjunctive coil embolization represents a safe alternative option for the treatment of IAs. In these cases, coil embolization increases the occlusion rate in PED flex-treated patients without increasing the periprocedural complications.

## Introduction

Flow diversion (FD) techniques, such as the pipeline embolization device (PED), have been largely accepted as important treatment options for large and complex intracranial aneurysms (IAs) (1–3). Similar to other flow diversions, PED represents a novel concept of curing aneurysms by redirecting the blood flow and reconstructing the parent vessel. Compared with conventional endovascular techniques (coiling or stenting), PED provides better neck reconstruction and contributes to complete occlusions (1, 4–6). Unfortunately, the rate of hemorrhagic complications caused by PED is reported to be up to 4%, especially among patients with large and giant aneurysms (4, 7), which may bring life-threatening disasters to the patients.

The underlying mechanisms leading to hemorrhage after PED deployment is complex and unclear yet (2, 8–10). Adjunctive coil embolization is recommended with PED to protect the aneurysm dome (2, 11). However, the safety and efficacy of this endovascular technique has not been fully defined. The pipeline flex embolization device (PED flex), a second-generation of PED, has been clinically available and widely adopted in the treatment of aneurysms recently. Thus, the aim of our study was to assess the safety and efficacy of the PED flex together with coil embolization in the treatment of IAs.

## Materials and Methods

### Study Design and Patient Selection

This study was conducted by our institution, and the ethics committee of the institute approved the study protocol. A consecutive series of patients with intracranial aneurysms treated by PED flex has been maintained in our center. Medical records from January 2018 to June 2020 were retrospectively reviewed to collect the clinical and radiologic data of patients.

Patients whose treatment included PED flex were enrolled in our study. Routine angiographic follow-up was performed post-treatment. The exclusion criteria were as follows: (1) patients who were lost in the follow-up time, which made the clinical data insufficient, (2) patients who were followed up in other hospitals, and (3) patients who were treated with any other stent when the PED flex was deployed at the same time. The study cohort was subdivided into two subgroups: one group included patients treated only through PED alone, and the other group included patients treated through PED flex adjunctive with coil.

### Treatment Strategy

Informed consents were provided by all patients or their relatives before endovascular treatment. All procedures were conducted under general anesthesia. Premedication usually consisted of dual antiplatelet management of aspirin (100 mg) and clopidogrel (75 mg) once a day for 5–7 days before the procedure. The antiplatelet resistance test was performed in all patients 1 day prior to the procedure. Those who underwent endovascular treatment on emergencies received a loading dose of tirofiban (10 m) prior to the endovascular procedure, which was maintained with a dosage of 6–8 ml/h intraoperatively. An intravenous heparin infusion was taken continuously in case of the potential thromboembolic events during the operation.

PED flex was selected based on the diameter of the parent vessel and the morphological characteristics of the aneurysm. In detail, the aneurysm was large in size (>10 mm), with significant morphological irregularity, or with a wide neck (>4 mm). To maximize flow diversion, we attempted to match the size of the nominal PED flex diameter relative to the diameter of the parent vessel and the landing zone. The decision of whether to use coils in combination with PED flex was considered seriously and decided by the discretion of the interventionists in specific scenarios. In detail, (1) when an aneurysm ruptured or had a high risk of precursory rupture due to the irregular morphological features—daughter aneurysms, for example; (2) when the aneurysm was large (>10 mm) or giant (>25 mm); and (3) when the patients had obvious clinical symptoms, such as serious headache or oculomotor nerve paralysis.

All procedures were performed *via* a standard transfemoral approach. A triaxial system with a guiding catheter, an intermediate catheter, and a marksman microcatheter was used to deliver the PED flex. After positioning the microcatheter distal to the aneurysmal neck, a second microcatheter paralleling to the intermediate catheter was navigated into the aneurysmal sac to subsequently deploy coils. According to previous reports, dense coil packing could cause a mass effect on the PED and result in device thrombosis (11, 12). Thus, we placed limited coils to make loose coiling embolization after the deployment of PED flex.

After the completion of endovascular treatment, all patients were kept on dual-antiplatelet medication for at least 6 months. Subsequently, the regimen was changed to aspirin monotherapy, which was continued for their whole life. Raymond classification was used to assess the angiographic results immediately after treatment and during follow-up (13). The radiological outcomes were classified as satisfactory (Raymond I and II) and poor (Raymond III).

### Statistical Analysis

Statistical analysis was performed with statistical software (SPSS v19.0; IBM, Chicago, IL, USA). One-sample Kolmogorov–Smirnov test was used to test the normal distribution for continuous variables, followed by independent-samples *t*-test of approximately normally distributed data. The Mann–Whitney *U*-test was used to compare non-parametric data, while χ^2^ test was used for categorical variables. The categorical variables were presented as frequencies, and the continuous variables were presented as mean ± SD or median (interquartile range). A *P* < 0.05 was considered statistically significant.

## Results

A total of 200 patients were treated with PED flex in our institute from January 2018 to June 2020. We excluded a total of 75 patients, eight of them due to loss of follow-up and the remaining 67 patients due to follow-up in other hospitals. Therefore, our patient cohort included 125 patients with 140 IAs that underwent PED flex placement with/without adjunctive coiling. Of these, 41 aneurysms were treated by PED flex plus coils (PED flex + coil group), while the other 99 aneurysms were treated with PED flex alone (PED flex group).

### Baseline Characteristics of Patients

The patient demographic and clinical characteristics are shown in Table 1. There was no statistically significant difference between the PED flex group and the PED flex + coil group in age, sex, smoking, drinking, basic diseases, and clinical symptoms (*P* > 0.05, Table 1). The most frequent clinical presentation in PED flex + coil group patients was headache (13/41, 31.7%), whereas the majority of the PED flex group patients were asymptomatic (Table 1).

**Table 1:** Demographic and clinical characteristics of patients.

	**PED flex (*n* = 99)**	**PED flex+coil (*n* = 41)**	***P***
Age (mean ± SD, year)	53.3 ± 9.5	53.2 ± 10.0	0.068
Female sex (*n*, %)	73 (73.7%)	32 (78.0%)	0.592
Smoking (*n*, %)	13 (13.1%)	6 (14.6%)	0.813
Drinking (*n*, %)	11 (11.1%)	4 (9.8%)	1
Basic disease (*n*, %)			
Hypertension	46 (46.5%)	20 (48.8%)	0.803
Diabetes	10 (10.1%)	3 (7.3%)	0.606
Hyperlipidemia	11 (11.1%)	6 (14.6%)	0.767
Symptom (*n*, %)			
Asymptomatic	31 (31.3%)	11 (26.8%)	0.598
Dizziness	27 (27.3%)	10 (24.4%)	0.725
Headache	24 (24.2%)	13 (31.7%)	0.362
Other	17 (17.2%)	7 (17.1%)	0.825

### Aneurysm Characteristics

The mean aneurysm size was 6.0 ± 4.3 mm in the PED flex group, with a mean width of 5.1 ± 3.4 mm, and 10.0 ± 5.8 mm in the PED flex + coil group, with a mean width of 7.2 ± 4.6 mm. A larger size of aneurysms was observed in the PED flex + coil group than that in the PED group (10.0 ± 5.8 vs. 6.0 ± 4.3 mm, *P* < 0.001, Table 2). Aneurysms in the PED flex +coil group were likewise wider than those in the PED flex group (7.2 ± 4.6 vs. 5.1 ± 3.4 mm, *P* = 0.002, Table 2). In both groups, the majority of aneurysms were located in the anterior circulation, which had no statistical significance (89.9 vs. 90.2%, *P* > 0.05, Table 2). Additionally, the number of aneurysms with a side branch coming from the sac in the two groups was similar (7.1 vs. 7.3%, *P* > 0.05, Table 2). A higher proportion of ruptured aneurysms was observed in the PED flex + coil group, although there was no statistical difference between the two groups (*P* = 0.355, Table 2).

**Table 2:** Aneurysm characteristics.

	**PED flex (*n* = 99)**	**PED flex+coil (*n* = 41)**	***P***
Size (mean ± SD, mm)	6.0 ± 4.3	10.0 ± 5.8	<0.001^*^
Width (mean ± SD, mm)	5.1 ± 3.4	7.2 ± 4.6	0.002^*^
Anterior circulation (*n*, %)	89 (89.9%)	37 (90.2%)	1
ICA	84	36	1
MCA	2	1	
ACA	3	0	
With side branch	7 (7.1%)	3 (7.3%)	
Rupture (*n*, %)	4 (4.0%)	4 (9.8%)	0.355

*ICA, internal carotid artery; MCA, middle cerebral artery; ACA, anterior cerebral artery*.

* *P < 0.05*.

### Treatment-Related Factors

In our cohort, PED flex deployment and coil embolization were successful in all patients. The periprocedural complications, angiographic results of initial postoperative and follow-up, and intervals of follow-up are summarized in Table 3.

**Table 3:** Treatment-related factors.

	**PED flex** **(*n* = 99)**	**PED flex+coil** **(*n* = 41)**	***P***
Periprocedural complication (*n*, %)	1 (1.0%)	2 (4.9%)	0.205
Initial angiography (*n*, %)			<0.001^*^
Complete occlusion	0(0%)	15 (36.6%)	
Residual neck	0(0%)	2 (4.9%)	
Residual aneurysm	99 (100%)	24 (58.5%)	
Follow-up angiography (*n*, %)			0.137
Complete occlusion	71 (71.7%)	35 (85.4%)	
Residual neck	7 (7.1%)	3 (7.3%)	
Residual aneurysm	21 (21.1%)	3 (7.3%)	
Satisfactory angiography results	78 (78.8%)	38 (92.7%)	0.047^*^
Follow-up time (mean ± SD, month)	6.2 ± 1.7	6.2 ± 1.8	0.998

* *P < 0.05*.

One periprocedural complication occurred in a patient who received PED flex, while two complications were observed in subjects who received additional coiling. All complications were considered ischemic events, which were mainly caused by the poor wall apposition of the PED flex that resulted in significant in-stent stenosis rather than the coil embolization technique itself. The percentages of perioperative complications in both groups were statistically comparable (1.0 vs. 4.9%, *P* = 0.205, Table 3). No patient suffered adverse events during the follow-up period.

**Figure 1:**
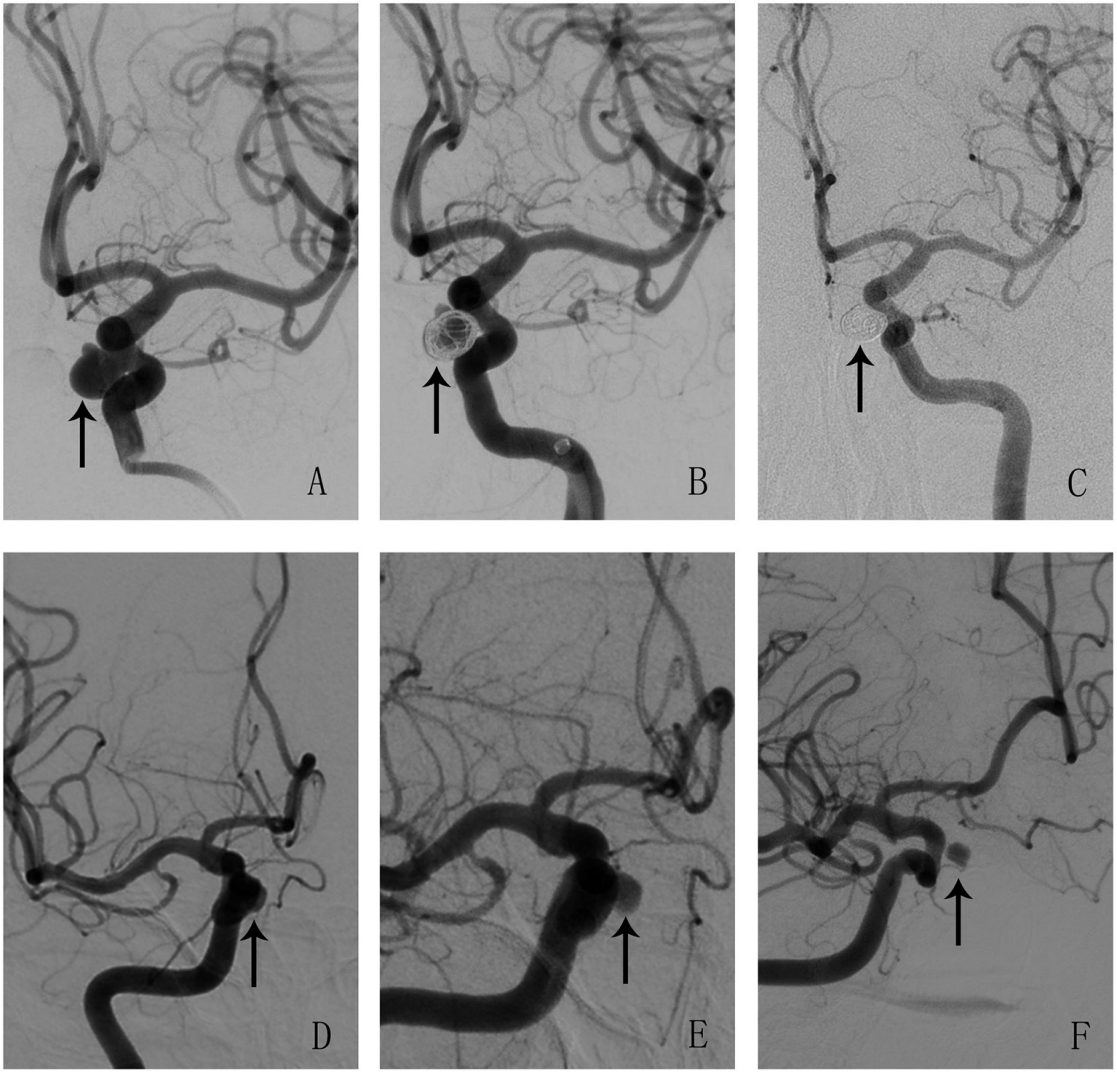
Illustrations of angiographic results in a case treated by pipeline flex embolization device (PED flex) adjunctive with coils (**A–C**) and a case treated by PED flex alone (**D–F**). Aneurysms were pointed out with arrows. (**A–C**) An aneurysm treated by PED + coil at pre-treatment, post-treatment, and follow-up. The angiography results showed that the aneurysm was cured completely after operation at 6 months. (**D–F**) An aneurysm treated by PED flex at pre-treatment, post-treatment, and follow-up. This aneurysm was incompletely occluded and was classified as Raymond III at 6 months.

In the PED flex group, all aneurysms presented remarkable stagnation, graded as Raymond III, as indicated from the immediate postoperative angiograms. Conversely, patients who received PED flex + coil had better immediate angiographic results, with 15 cases (36.6%) showing complete occlusion (Raymond I) and two cases (4.9%) showing residual aneurysms (Raymond II). Overall, the initial angiographic results of the two groups were statistically different (*P* < 0.001, Table 3). The mean follow-up interval after surgery was 6.2 ± 1.7 months for the PED flex group and 6.2 ± 1.8 months for the PED flex + coil group, respectively. The total complete occlusion rate was 75.7% during follow-up. Aneurysms treated by PED + coil could be completely occluded nearly 6 months after surgery, while the aneurysms remained Raymond III when treated by PED flex alone (Figure 1). A higher rate of complete occlusion was achieved in the PED flex + coil group at follow-up (85.4 vs. 71.7%, respectively, Table 3). However, the whole angiography result classifications on follow-up at 6.2 months were comparable (*P* = 0.137, Table 3). Of note is that a higher percentage of aneurysms presented with satisfactory angiography results (Raymond class I and II) in the PED flex + coil group during follow-up (92.7 vs. 78.8%, *P* = 0.047, Table 3).

## Discussion

The flow diverter is a milestone in the treatment of IAs despite unpredictable complications. To prevent undesired hemorrhagic outcomes, coils in conjunction with PED became an alternative treatment option gradually (12, 14). However, before being widely accepted and used as a treatment modality, this technique therapy requires an understanding of its benefits and potential disadvantages. Therefore, we report our single-center experience of the concomitant use of PED flex in adjunction with coil embolization and determine its feasibility, safety, and effectiveness by comparing to PED flex embolization as standalone technique.

Our data demonstrated that aneurysms treated by PED flex-assisted coils tend to be larger and wider. For these aneurysms, using PED flex alone may need a longer time to achieve complete occlusion, posing a significant risk of rupture to the patients (15). As shown in our cohort, the concurrent use of coils is clearly beneficial and safe. We did not experience fatal complications such as hemorrhage or delayed aneurysmal rupture, with no statistical difference of periprocedural complication rate within both treatment groups. To our knowledge, there is no consensus on the indications for coils adjunctive with PED flex, and the underlying mechanism of coils sparing aneurysms from rupture in PED flex-deployed cases remains unknown. Studies based on computational flow dynamics have quantitatively illustrated the critical effect of intra-aneurysmal pressure and flow velocities on the prognosis of the aneurysm (16, 17). It has been reported that the mere application of FD may only reduce relatively low the amplitude and pressure despite significant flow velocity changes inside the aneurysms (16, 18, 19). Thus, a combination of PED flex and coils may become a suggestive way of modifying the blood velocities and pressures distinctly at the same time. Additionally, the use of coils may also elicit a biological effect. An animal study conducted by Evan et al. demonstrated that concomitant coiling could reduce the level of active-MMP9 in FD-treated aneurysms by blocking the activation of pro-MMP9 (20). MMPs, whose expression was regulated by the adjunctive use of coils, played key roles in delayed ruptures after FD deployment. Moreover, the effective use of PED flex along with coils could avoid the technical difficulty and challenge of deploying multiple PEDs, lowering the risk of potential ischemic events (4, 15, 21).

It is worth to mention that all the periprocedural complications in our cohort are non-hemorrhagic events, which may correlate with thrombosis closely. Intravascular deployment of PED stimulates platelet aggregations as soon as it is exposed to the blood. As reported, the wall apposition of PED and the management of antiplatelets are of great value for preventing thrombotic events (11, 12, 21, 22). Therefore, in addition to the intraprocedural anticoagulation, prophylactic antiplatelet therapy should be executed strictly before and after the procedure. Excitingly, coil packing following PED flex is technically easy to achieve, and there was no technique-related complication regarding coiling in our study.

Similar to previous studies, our present results demonstrated that coiling in conjunction with PED could achieve a higher occlusion rate for certain IAs (11, 23, 24). It is known that coils were initially designed to pack the aneurysm tightly, thereby preventing blood from flowing into the aneurysm and protecting the aneurysm from growing. Multiple mechanisms account for the improved occlusion results by the combination of coils and PED flex, mainly including hemodynamic changes and thrombosis. One possibility is that the coils contribute to the thrombosis inside the aneurysm, acting as a foreign body material and activating inflammatory responses (25–27). Another possibility is that the flow hemodynamics, especially velocities and wall shear stress, which are changed profoundly with the implantation of coils, is conducive to neointima formation on the aneurysm orifice (16, 27).

Overall, coils adjunctive with PED flex are complementary, rather than competing, for cerebral aneurysm treatment (11). On the one hand, coils can serve as an essential architecture to protect the PED flex from herniating into the aneurysm, thus avoiding shortening or poor wall apposition of the flow diverter. On the other hand, PED flex acts as a scaffold to prevent the coils from prolapsing into the parent vessel. Although randomized controlled trials comparing PED flex alone and PED flex adjunction with coiling are not available yet, coils in conjunction with PED flex are likely to play a dominant role in improving the occlusion rate of aneurysms.

## Conclusion

PED flex placement with adjunctive coil embolization represents a safe alternative option for the treatment of IAs. In these cases, coil embolization increases the occlusion rate at an early stage without increasing the periprocedural complications. Further clinical and basic studies are needed to identify the impact and role of coils in PED flex-treated cases to establish treatment guidelines.

### Limitation

There are some potential limitations in our current study. First, this is a retrospective single-center study with a relatively small case series; thus, multicenter studies with a larger number of patients are required for in-depth analysis. Second, our reports are mainly based on short-term follow-up time points, and the prognosis of aneurysm may evolve into other acceptable results over time. Longer temporal follow-up is needed to point out the potential complications and adverse effects. Besides this, the treatment strategy for which aneurysm was warranted adjunctive coiling was determined at the discretion of the attending surgeon. In the future, further randomized researches are supposed to be performed in order to provide stronger evidence to support these preliminary results.

## Data Availability Statement

The original contributions presented in the study are included in the article/supplementary material, further inquiries can be directed to the corresponding authors.

## Ethics Statement

The studies involving human participants were reviewed and approved by Henan Provincial People's Hospital. The patients/participants provided their written informed consent to participate in this study.

## Author Contributions

QZ contributed to the preparation of the manuscript and data collection. KC, HZ, XM, and QS contributed to data analysis and interpretation. ZS, HA-B, and AZ contributed to the editing and revision of the manuscript. YH and LL contributed to the revision of the manuscript. LL and TL contributed to the study design. All authors contributed to the article and approved the submitted version.

## Funding

This work was supported by the National Key Research and Development Program of China (2016YFC1300702), the Funding Plan for Key Scientific Research Projects of Colleges and Universities in Henan Province (21A320002), and the Henan Province Young and Middle-Aged Health Science and Technology Innovation Talent Training Project (YXKC2020041).

## Conflict of Interest

The authors declare that the research was conducted in the absence of any commercial or financial relationships that could be construed as a potential conflict of interest.

## Publisher's Note

All claims expressed in this article are solely those of the authors and do not necessarily represent those of their affiliated organizations, or those of the publisher, the editors and the reviewers. Any product that may be evaluated in this article, or claim that may be made by its manufacturer, is not guaranteed or endorsed by the publisher.
